# VIP Modulation of Hippocampal Synaptic Plasticity: A Role for VIP Receptors as Therapeutic Targets in Cognitive Decline and Mesial Temporal Lobe Epilepsy

**DOI:** 10.3389/fncel.2020.00153

**Published:** 2020-06-12

**Authors:** Diana Cunha-Reis, Ana Caulino-Rocha

**Affiliations:** ^1^BioISI - Biosystems and Integrative Sciences Institute, Faculdade de Ciências, Universidade de Lisboa, Lisbon, Portugal; ^2^Departamento de Química e Bioquímica, Faculdade de Ciências, Universidade de Lisboa, Lisbon, Portugal

**Keywords:** VIP, synaptic plasticity, interneurons, hippocampus, MTLE, cognition, VPAC1 receptors

## Abstract

Vasoactive intestinal peptide (VIP) is an important modulatory peptide throughout the CNS acting as a neurotransmitter, neurotrophic or neuroprotective factor. In the hippocampus, a brain area implicated in learning and memory processes, VIP has a crucial role in the control of GABAergic transmission and pyramidal cell activity in response to specific network activity by either VIP-containing basket cells or interneuron-selective (IS) interneurons and this appears to have a differential impact in hippocampal-dependent cognition. At the cellular level, VIP regulates synaptic transmission by either promoting disinhibition, through activation of VPAC_1_ receptors, or enhancing pyramidal cell excitability, through activation of VPAC_2_ receptors. These actions also control several important synaptic plasticity phenomena such as long-term potentiation (LTP) and long-term depression (LTD). This paper reviews the current knowledge on the activation and multiple functions of VIP expressing cells in the hippocampus and their role in controlling synaptic transmission, synaptic plasticity and learning and memory processes, discussing also the role of VPAC_1_ and VPAC_2_ VIP receptors in the regulation of these different processes. Furthermore, we address the current knowledge regarding changes in VIP mediated neurotransmission in epileptogenesis and mesial temporal lobe epilepsy with hippocampal sclerosis (MTLE-HS), and discuss the therapeutic opportunities of using selective VIP receptor ligands to prevent epileptogenesis and cognitive decline in MTLE-HS.

## Introduction

Vasoactive intestinal peptide (VIP), a 28 amino-acid residue peptide originally isolated from porcine duodenum by Mutt and Said (1974), owes its name to its powerful ability to cause vasodilatation (Said and Mutt, 1970), by promoting vascular smooth muscle relaxation in the gastrointestinal tract when released by peripheral nerves of the sympathetic nervous system (Said and Rosenberg, 1976). In subsequent years, VIP was described in multiple peripheral and central neuronal control systems, where it acts as neurotransmitter, neurotrophic or neuroprotective factor (Borbély et al., 2013; Deng and Jin, 2017). Discovery of pituitary adenylate cyclase-activating polypeptide (PACAP) in the ovine hypothalamus (Miyata et al., 1989), where it acts as an endocrine regulator, brought additional complexity to the understanding of the actions of VIP, since these two peptides share common receptors and are often present together in the same brain regions (see below). The actions of PACAP on synaptic transmission, plasticity and cognition are reviewed in another paper in this research topic (Ciranna and Costa, 2019) and will be discussed here only when clarifying the duality of VIP vs. PACAP signaling. VIP is nowadays recognized as an important modulator of synaptic transmission and plasticity, network excitability as well as of learning and memory processes and has been associated with cognitive deficits in several central nervous system (CNS) diseases. This paper reviews the multiple roles of VIP in synaptic transmission, synaptic plasticity and hippocampal-dependent learning and memory processes, the role of VIP in hippocampal and cognitive disfunction in mesial temporal lobe epilepsy (MTLE) and the therapeutic opportunities that this presents.

## VIP in the Hippocampus

Upon its discovery, VIP expression was reported in the human hippocampus and the hippocampus of animal models (Emson et al., 1979; Lorén et al., 1979; Besson et al., 1984), where VIP was also shown to bind to hippocampal membranes (Taylor and Pert, 1979; Besson et al., 1984). Shortly after, it became evident that VIP expression was predominant in hippocampal GABAergic interneurons (Köhler, 1982, 1983; Léránth et al., 1984; Kosaka et al., 1985) and that modulation of GABAergic transmission was likely an important target for VIP action. VIP was also early recognized to have a crucial role in mnemonic processes and particularly in hippocampal-dependent memory traits (Cottrell et al., 1984; Flood et al., 1990; Glowa et al., 1992). Nevertheless, the first report of its physiological actions in the CNS described VIP excitation of hippocampal CA1 neurones (Dodd et al., 1979). This enhancement in pyramidal cell excitability was latter shown to occur essentially through reduction of the Ca^2+^- and cAMP-dependent K^+^-conductance, leading to a decrease of the long-lasting afterhyperpolarization (sAHP) and a reduction of the accommodation of firing (Haas and Gähwiler, 1992). This action was postsynaptic since it prevailed in low Ca^2+^ – high Mg^2+^ medium and was later demonstrated to depend on protein kinase A (PKA) activity (Haug and Storm, 2000). Later, the actions of VIP on hippocampal GABAergic transmission were described showing that VIP increases the frequency of miniature IPSCs in cultured pyramidal neurones without affecting their amplitude (Wang et al., 1997), which suggests a presynaptic facilitation of GABA release by VIP. This appeared contradictory since VIP actions would lead to opposing effects on pyramidal cell excitability. All these findings are summarized in Table 1. When the anatomy of VIP-expressing interneurons (*VIP^+^ INs*) in the hippocampus was elucidated (Acsády et al., 1996a, b; Hájos et al., 1996) the different roles of VIP in modulation of hippocampal GABAergic transmission and regulation of pyramidal cell excitability began to be clarified.

**TABLE 1:** Effects of VIP on hippocampal excitatory and inhibitory networks and VIP receptors involved.

Action	Target	Receptor	Species/preparation	References
Enhanced pyramidal cell excitability	CA1 PNs	Unknown	Rat hippocampal slices	Dodd et al., 1979
Enhanced synaptic transmission and pyramidal cell excitability and; Reduced the slow afterhyperpolarization (Ca^2+^-dependent K^+^ current)	CA1 PNs	Unknown	Male Wistar rats (young adult): hippocampal slices	Haas and Gähwiler, 1992
VIP application to the *O/A* in the absence of synaptic interactions, increased the firing rate O/A INs and; Decreased fEPSP slope rat the *SR* and *SLM*,	CA1 INs and PNs	Unknown	Male NMRI mice (young adult): hippocampal slices	Yanovsky et al., 1997
Increased the frequency of mIPSCs without affecting their mean magnitude	Hippocampal neurons	Unknown	Cultured hippocampal neurons	Wang et al., 1997
Enhanced EPSCs	CA1 PNs	Unknown	Juvenile male Wistar rats: hippocampal slices	Ciranna and Cavallaro, 2003
Enhanced synaptic transmission through disinhibition and pyramidal cell excitability Enhanced GABAergic currents Enhanced GABA release	CA1 PNs (dendrites and soma) CA1 INs and PNs GABAergic nerve terminals	Unknown	Male Wistar rats (young adult): hippocampal slices	Cunha-Reis et al., 2004
VIP enhanced synaptic transmission	CA1 PNs (dendrites)	VPAC_1_ and VPAC_2_ receptors	Male Wistar rats (young adult): hippocampal slices	Cunha-Reis et al., 2005
Enhances pyramidal cell excitability	CA1 PNs	VPAC_2_ receptor	Male Wistar rats (young adult): hippocampal slices	Cunha-Reis et al., 2006
VIP enhanced the amplitude of NMDARs	CA1 PNs	VPAC_1_/VPAC_2_ receptors	Juvenile and young adult male wistar rats: isolated CA1 neurons and hippocampal slices	Yang et al., 2009
Endogenous VIP inhibits CA1 hippocampal LTP	CA1 PNs (dendrites)	VPAC_1_ receptor	Male Wistar rats (young adult): hippocampal slices	Cunha-Reis et al., 2010
Endogenous VIP inhibits hippocampal CA1 LTD and depotentiation	CA1 PNs (dendrites)	VPAC_1_ receptor	Juvenile and young adult male Wistar rats: hippocampal slices	Cunha-Reis et al., 2014
Enhances exocytotic GABA release and GAT-1 nerve terminal reversal Inhibits exocytotic GABA release	GABAergic nerve terminals	VPAC_2_ receptor VPAC_1_ receptor	Male Wistar rats (young adult): isolated nerve terminals	Cunha-Reis et al., 2017

*CA1, hippocampus Cornus Ammonis 1; GAT-1, GABA transporter 1; INs, interneurons; NMRI, Naval Medical Research Institute; O/A, hippocampal Oriens-Alveus layer; PNs, Pyramidal neurons; SR, Stratum radiatum; SLM, Stratum lacunosum-moleculare; VPAC_1_ and VPAC_2_, VIP – Vasoactive intestinal peptide; VIP receptors 1 and 2.*

**FIGURE 1:**
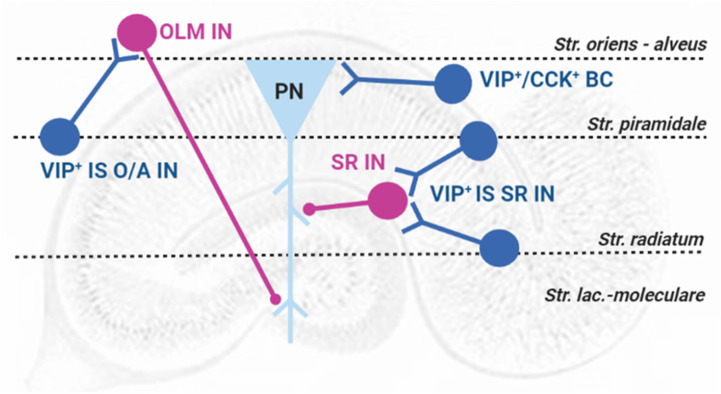
Representation of VIP-containing interneurons in the rat hippocampus: layer location and target selectivity. PN, pyramidal neuron (triangle, light blue); Interneurons (circles, pink); VIP-containing interneurons (circles, blue); *VIP^+^-CCK^+^ BCs*: VIP-containing *basket cells*; *VIP^+^ IS O/A IN*: VIP-containing interneuron-selective interneuron targeting the *stratum oriens*/*Alveus* and *VIP^+^ IS SR IN*: VIP-containing interneuron-selective interneuron targeting the *stratum radiatum*; OLM IN – *Stratum oriens* interneuron projecting to the *Stratum lacunosum-moleculare*; SR IN – *Stratum radiatum* local interneurons. Str.: *stratum.*

Detailed immunohistochemistry studies fully characterized hippocampal *VIP^+^ INs* dendritic trees and axon projections (Acsády et al., 1996a, b), allowing the classification of *VIP^+^ INs* into two fundamental groups according to their targets: *VIP^+^ basket cells* are responsible for somatic inhibition of pyramidal cells, are also immunoreactive for cholecystokinin (*VIP^+^-CCK^+^ BCs*, Figure 1) and do not express parvalbumin, as most *BCs* in the hippocampus. *VIP*^+^ *INs* that selectively innervate other interneurons (*VIP*^+^ *IS INs*) include two subtypes: (a) interneurons with cell bodies located at the *stratum pyramidale (SP)* or near and projecting to the *stratum Oriens/Alveus* border (*VIP*^+^ *IS O/A INs* or type III IS cells, Figure 1), that also express the interneuron marker calretinin and target mostly somatostatin-expressing (SOM^+^) *oriens lacunosum-moleculare* (*OLM*) interneurons innervating the distal dendrites of pyramidal cells at the *stratum lacunosum-moleculare (SLM)* and (b) *VIP*^+^ *INs* that project their axons to the *stratum radiatum* (*SR*, *VIP^+^ IS SR INs*, Figure 1), with cell bodies located either at the *SR/SLM* border (type II IS cells) or at *SR/SP* and targeting *interneurons* controlling synaptic transmission to proximal dendrites of pyramidal cells in the *SR* (Acsády et al., 1996a, b; Klausberger and Somogyi, 2008). In genetically modified VIP-eGFP mice, additional targets of *VIP^+^ IS O/A INs* in the *O/A*, including bistratified cells and oriens–oriens INs, have been described and recently a new VIP expressing interneuron population located at the O/A (*VIP*^+^ long-range projecting INs, *VIP*^+^ *LRP INs*) was described targeting *INs* within the O/A in CA1 but also both *INs* and pyramidal cells within the *subiculum* (Francavilla et al., 2018). It is not clear if it is also present in the rat hippocampus.

Considering the early acquired knowledge (Acsády et al., 1996a, b) on the target selectivity of VIP-IS hippocampal interneurons in the rat, Yanovsky et al. (1997) studied the influence of VIP application to the *Oriens/Alveus* border and showed that in the absence of synaptic interactions, VIP increased the firing rate of these interneurons and decreased the slope of the fEPSPs recorded at the *SR* and *SLM*, thus decreasing excitatory synaptic transmission through an increase in inhibitory transmission (Yanovsky et al., 1997). These mechanisms could not account for the previously observed increase in synaptic transmission and pyramidal cell firing (Haas and Gähwiler, 1992). In fact, VIP-mediated concomitant pre and post-synaptic enhancement of GABAergic transmission generating disinhibition of synaptic transmission to pyramidal cell dendrites (Cunha-Reis et al., 2004) appears to coexist with direct VIP mediated actions on pyramidal cell bodies either promoting enhancement glutamatergic EPSCs (Ciranna and Cavallaro, 2003) or GABAergic currents (Cunha-Reis et al., 2004) in rat hippocampal slices. The physiological relevance of these conflicting observations remains poorly understood but may be relevant in distinct physiological conditions, depending on network and behavioral state-dependent activation of different interneuron populations (Tyan et al., 2014; Artinian and Lacaille, 2018; Francavilla et al., 2018; Turi et al., 2019; Luo et al., 2020).

## VIP and VIP Receptors

VIP shows structural similarity to other neuroendocrine peptides, including secretin, glucagon, gastric inhibitory peptide, growth hormone releasing factor and PACAP (Deng and Jin, 2017); and belongs glucagon–secretin–VIP family of peptides targets (Clynen et al., 2014). VIP acts trough two high affinity receptors (VPAC_1_ and VPAC_2_) that belong to Group II receptor (GPCR) family and are encoded by two different genes sharing only 55% similarity. These have nearly the same affinity for VIP (in the low nanomolar range) (Yang et al., 2010; Harmar et al., 2012) and bind also PACAP with similar affinity, hence the name VPAC given to VIP receptors (Laburthe et al., 2002). The VIP receptor subfamily also includes a third receptor, PAC_1_ (PACAP specific receptor), which binds VIP with low affinity (in the micromolar range) (Harmar et al., 2012). VPAC receptors exhibit multiple consensus sites for phosphorylation by intracellular kinases and *N*-glycosylation, but differences in the *N*-glycosylation are observed according to tissue and/or species (Laburthe et al., 2002).

Both VPAC and PAC_1_ receptors are positively coupled to G_αs_ and stimulate the cAMP/PKA signaling pathway (see Laburthe et al., 2002; Harmar et al., 2012 for review). However, PAC_1_ receptors additionally strongly stimulate G_αq_ and the phospholipase C (PLC)/PKC signaling pathway, while VPAC receptors activate it weakly (Harmar et al., 1998; Yang et al., 2010). However, VPAC_1_ receptors can couple to G_i/o_ proteins in the hippocampus (Shreeve, 2002) and VIP enhancement of [Ca^2+^]_i_ in astrocyte cultures depends on IP_3_ turnover (Fatatis et al., 1994).

## VIP Receptors and Hippocampal Neurotransmission

Although VIP and PACAP receptors have a widespread expression in the brain, VPAC_1_ receptors are predominantly found in the hippocampus and cerebral cortex, while VPAC_2_ receptors prevail in the thalamus and suprachiasmatic nucleus, showing lower expression in the hippocampus, spinal cord, dorsal root ganglia and brainstem (Harmar et al., 2012; Borbély et al., 2013). Not surprisingly, VIP and VIP receptors are involved in learning and memory processes (Yang et al., 2010; Borbély et al., 2013), yet, elucidating the differential involvement of each VIP receptor in the actions of VIP has proven very difficult until ligands with enough selectivity to discriminate between VPAC_1_ and the VPAC_2_ receptor were developed (Gourlet et al., 1997a,b,c; Moreno et al., 2000). This is particularly important in the hippocampus, where both receptors are expressed (Vertongen et al., 1997; Joo et al., 2004).

VIP receptors are unevenly distributed in different hippocampal layers. VPAC_2_ receptors are more expressed in *SP* of the Ammon’s Horn implying a key role in the modulation of hippocampal pyramidal cell activity, whereas VPAC_1_ receptors are preferentially located in the *SO* and *SR* and partially co-localized with glial markers (Acsády et al., 1996a; Joo et al., 2004). No study has to date identified VPAC_1_ receptors in hippocampal interneurons, yet the fact that VIP enhancement of synaptic transmission to CA1 pyramidal cells involves inhibition of GABAergic interneurons that control pyramidal cell dendrites, leading to disinhibition (Cunha-Reis et al., 2004), an action mediated by activation of VPAC_1_ receptors (Cunha-Reis et al., 2005) preferentially located in the *SO*, *SR* or *O/A* (Vertongen et al., 1997; Joo et al., 2004) suggests VPAC_1_ receptors are in fact responsible for VIP actions on hippocampal interneurons. VPAC_2_ receptors are the main mediators of VIP enhancement of pyramidal cell excitability (Cunha-Reis et al., 2006), and likely mediators of VIP enhancement of NMDA receptor currents in pyramidal cells (Yang et al., 2009), effects that are mostly post-synaptic and independent of GABAergic transmission (Ciranna and Cavallaro, 2003; Cunha-Reis et al., 2004), and that likely involve inhibition of the sAHP (Haas and Gähwiler, 1992) (see Table 1).

VIP modulation of hippocampal GABAergic transmission involves both presynaptic enhancement of GABA release and postsynaptic facilitation of GABAergic currents in interneurons (Wang et al., 1997; Cunha-Reis et al., 2004). We recently reported a dual opposing regulation of GABA release by VPAC receptors in isolated hippocampal nerve terminals (Cunha-Reis et al., 2017): VPAC_1_ receptors inhibit and VPAC_2_ receptors enhance GABA release. VPAC_1_ receptor activation inhibits voltage-gated calcium channel (VGCC)-dependent GABA exocytosis through a G_i/o_ and PKA-independent and partially PKC-dependent mechanism (Cunha-Reis et al., 2017). VPAC_2_ receptor activation enhances VGCC-dependent GABA exocytosis by a G_s_/PKA/PKC-dependent mechanism but also enhances GAT-1 carrier-mediated GABA outflow through a G_s_/PKC-dependent mechanism. Given the asymmetry in VPAC_1_ and VPAC_2_ receptor location in different layers of Ammon’s horn, VIP may differentially modulate GABA release to pyramidal cells and *INs*, and thus have distinct consequences on synaptic transmission to pyramidal cell dendrites and pyramidal cell activity, suggesting several possible therapeutic applications.

## VIP and Synaptic Plasticity

Synaptic plasticity relies on long-lasting, activity-dependent bidirectional changes in the strength of synaptic communication leading to long-term potentiation (LTP) and long-term depression (LTD) (Mellor, 2018) and is widely accepted as the cellular mechanism underlying memory storage (Bliss and Collingridge, 2019). LTP can be triggered by a single episode of high frequency stimulations (HFS), such as a tetanus or theta burst (Albensi et al., 2007; Larson and Munkácsy, 2015; Bliss and Collingridge, 2019), mimicking the firing of hippocampal principal cells during learning tasks, and was the first synaptic plasticity mode to be associated with hippocampal-dependent memory formation (Bliss and Collingridge, 2019). LTD can be elicited by low-frequency stimulation (LFS), mimicking hippocampal activity during delta waves, and is involved in hippocampal-dependent memory processes associated with behavioral flexibility like memory extinction, reversal learning, reformulation of previously formed memories, terminating/shifting attention and in stabilizing the effects of learning (Kitchigina et al., 1999; Albensi et al., 2007; Kemp and Manahan-Vaughan, 2007; Collingridge et al., 2010). Both LTP and LTD require the activation of NMDA receptors, and their stability or long-lasting expression is dependent on subsequent activation of multiple intracellular cascades (Collingridge et al., 2010; Bliss and Collingridge, 2019). LTP of glutamatergic transmission requires activation of Ca^2+^/calmodulin-dependent protein kinase II (CaMKII) and recruitment and insertion of AMPA receptors into the postsynaptic membrane (early-LTP) (Park et al., 2018; Benke and Traynelis, 2019). Endurance and stability of LTP is believed to require synaptic contact enlargement and both PKA activity and *de novo* protein synthesis (late-LTP) (Park et al., 2018; Bliss and Collingridge, 2019).

Recent evidence supports the view that disinhibition plays a crucial role in regulating hippocampal synaptic plasticity (Artinian and Lacaille, 2018). Furthermore, Yang et al. (2009) showed that exogenously applied VIP enhances NMDA currents in CA1 pyramidal cells, an effect mimicked by VPAC_2_ and to a lesser extent by VPAC_1_ selective agonists. This suggests that either endogenous VIP or PACAP, the two endogenous agonists of this receptor, could contribute to NMDA-dependent hippocampal synaptic plasticity such as LTP, LTD and depotentiation. We recently described that endogenous VIP, through VPAC_1_ receptor activation, modulates the NMDA receptor-dependent LTD and depotentiation in the CA1 area of the hippocampus (Cunha-Reis et al., 2014). Furthermore, disinhibition achieved through inhibition of VPAC_1_ receptors was more efficient than blockade of GABA_A_–mediated transmission in revealing LTD, suggesting that *SR* interneurons are fundamental in restraining synaptic adaptations underlying expression of LTD. VPAC_1_ receptor activation by endogenous VIP also enhances hippocampal LTP induced by TBS, an action that is dependent on GABAergic transmission and involves phosphorylation of GluA1 AMPA subunit by CamKII, a fundamental mechanism for receptor synaptic recruitment (Cunha-Reis et al., 2010; Carmo and Cunha-Reis, 2011; Cunha-Reis and Carmo, 2011) (see Table 1). Activation of hippocampal VPAC_2_ (but not VPAC_1_) receptors also promotes phosphorylation of GluA1 at Ser845 (Toda and Huganir, 2015), a PKA target site that is implicated in LTP maintenance and late-LTP (Benke and Traynelis, 2019).

VIP and PACAP modulation of hippocampal principal cell activity targets (directly or indirectly) both the dendritic and somatic compartments implicating these peptides in regulation of both Hebbian and homeostatic plasticity (Wefelmeyer et al., 2016; Yee et al., 2017; Foncelle et al., 2018), yet the physiological and behaviorally relevant stimuli for this modulation are still largely uncovered. Recently, it was described that *VIP*^+^ *IS INs* are activated by both Schaffer collateral and commissural excitatory fibers, being recruited fundamentally during theta oscillations but not during fast ripples (Luo et al., 2020), suggesting a fundamental role in information gating during spatial navigation and memory encoding. Accordingly, *VIP*^+^ *IS INs* are targeted by *medium raphe* serotonergic and GABAergic projections and septal cholinergic fibers, fundamental for the pacing, engagement and suppression of hippocampal theta rhythm (Vinogradova et al., 1999; Borhegyi et al., 2004; Vandecasteele et al., 2014).

Release of large dense core vesicles containing neuropeptides is known to require high-intensity repetitive stimulation, unlike release of small synaptic vesicles containing fast transmitters such as glutamate or GABA (Ghijsen and Leenders, 2005). Firing of VIP-containing interneurons locked with theta rhythm may suffice to release endogenous VIP from hippocampal nerve terminals.

## VIP in Cognitive Processes

Early from its discovery, VIP was described to have a crucial role in mnemonic processes and particularly in hippocampal-dependent memory traits. In particular, endogenous VIP was implicated in spatial learning in the Morris water maze (Glowa et al., 1992; Takashima et al., 1993a; Itoh et al., 1994), avoidance learning in the T-maze (Flood et al., 1990) or the shuttle box, together with reduced rearing exploratory behavior (Cottrell et al., 1984; Takashima et al., 1993b), suggesting that VIP is mainly involved in regulating motivated learning behavior. VIP has lateralized effects on the modulation of exploratory behavior and passive avoidance learning (Ivanova et al., 2008, 2009) and anxiolytic and anti-depressive effects (Ivanova et al., 2014), and rescues deficits in hippocampal-dependent passive avoidance learning tasks in a rat model of depression. Recently, VIP-mediated hippocampal disinhibition of pyramidal cell activity was shown to play a crucial role in goal-directed spatial learning tasks (Turi et al., 2019).

VIP-KO mice show decreased expression of VPAC_2_ and to a lesser extent VPAC_1_ receptors together with strong circadian rhythm disruption and enhanced arousal and hyperactivity in the open-field test (Girard et al., 2006). Furthermore, VIP-deficient mice shows impaired recall and reversal learning in a fear conditioning test and deficits in social behavior (Chaudhury et al., 2008; Stack et al., 2008). VPAC_2_-KO mice display normal acquisition of fear conditioning, contextual and cued fear memory, but impaired extinction of cued fear memory (Ago et al., 2017).

VIP participates in the pathophysiology of several neurological disorders associated with cognitive disfunction, like depression (Ivanova et al., 2012), autism spectrum disorders, Alzheimer’s disease (AD), Parkinson’s disease (PD) and epilepsy (de Lanerolle et al., 1995; Hill, 2007; White et al., 2010). Due to its anti-apoptotic, anti-inflammatory and neuroprotective actions, VIP and its receptors constitute promising therapeutic targets in many of these pathologies (Gozes, 2001; Delgado et al., 2002; Yu et al., 2017).

## VIP, Seizures, and Epilepsy

Epilepsy is the most common, chronic neurological disease (Devinsky et al., 2018) and is characterized by the incidence of recurrent, unprovoked seizures with associated cognitive, psychological and social disturbances (Clynen et al., 2014; Devinsky et al., 2018). According to its underlying causes epilepsy is classified into genetic or idiopathic. More than 500 genes are associated with predisposition to develop epilepsy (Devinsky et al., 2018). Idiopathic epilepsy, has unknown causes but often follows several possible precipitating events such as head trauma, stroke, brain hypoxia, infectious/autoimmune diseases, tumors or childhood febrile seizures (Clynen et al., 2014).

Mesial temporal-lobe epilepsy with hippocampal sclerosis (MTLE-HS), the most prevalent form of symptomatic focal epilepsy, is a heavy burden for the healthcare system. Many MTLE-HS patients are refractory to treatment with multiple anti-epileptic drugs, and amygdalohippocampectomy surgery is the last intervention to prevent complex partial seizures (Kuang et al., 2014). Declarative memory deficits (Helmstaedter and Kockelmann, 2006) are also a hallmark of MTLE-HS, that can be further aggravated by hippocampal removal. Most MTLE cases are idiopathic and evidence suggests that precipitating events trigger epileptogenesis by generating aberrant synaptic plasticity/neuronal excitability, excitotoxicity, secondary non-convulsive *status epilepticus*, inflammation and generation of reactive oxygen species (ROS) (Devinsky et al., 2018; Rana and Musto, 2018), ultimately leading to occurrence of spontaneous recurrent seizures. MTLE-HS is characterized by hippocampal sclerosis, massive neuronal loss and severe astrogliosis (Thom, 2014). Enhanced neurogenesis initially drives formation of new neural pathways in epileptogenesis (Beck and Yaari, 2008), but is impaired in MTLE-HS chronic phase (Zhong et al., 2016). Impaired LTP, due to pathological saturation (Beck et al., 2000), is a major cause for cognitive impairment in MTLE-HS, but changes in input/output neuronal electrical properties (El-Hassar et al., 2007a) and inhibitory/excitatory balance also occur from early in epileptogenesis (El-Hassar et al., 2007b). New drug targets able to control seizures or preventing epileptogenesis are an urgent need (Clynen et al., 2014).

Neuropeptides, such as VIP, are stored in large dense-core granules and are released during the sustained high-frequency activity (5–40 Hz) occurring during epileptiform activity, being implicated in regulation of seizure susceptibility, constituting appealing targets for the development of new AEDs, potentially less susceptible to side-effects (Clynen et al., 2014).

VIP is an important regulator of hippocampal activity through both direct actions on pyramidal cell excitability (Haas and Gähwiler, 1992) and by regulating synaptic transmission and synaptic plasticity to pyramidal cell dendrites through disinhibition (Cunha-Reis et al., 2004, 2010, 2014; Cunha-Reis and Carmo, 2011; Luo et al., 2020), actions that have a major impact on hippocampal-dependent learning and memory formation (Turi et al., 2019).

In human MTLE-HS, an up-regulation in VIP receptors in the seizure focus (hippocampus) was linked to the loss of principal neurons (i.e., granule cells and pyramidal neurons) without changes in the pattern and distribution of *VIP*^+^ *INs* (de Lanerolle et al., 1995). Accordingly, an enhancement in *VIP*^+^ *INs* has been described in a mouse model of temporal lobe epilepsy (TLE) (King and LaMotte, 1988) and decreased dendritic but not somatic GABAergic inhibition has been implicated in different animal models of experimental TLE (Sloviter, 1987; Cossart et al., 2001). Although an enhancement in disinhibition caused by VIP could be implicated in reduced seizure threshold in MTLE-HS, enhancement in VIP expression is more likely a compensatory mechanism for the selective loss of OLM interneurons in TLE, the main targets of *VIP^+^ IS O/A INs*. Recently, it was described that while the overall density of the *VIP^+^ IS O/A INs* was preserved, the number of their synaptic contacts in CA1 *O/A* was reduced in the pilocarpine model of TLE and was accompanied by significant alterations in their dendritic morphology and passive membrane properties (David and Topolnik, 2017).

Following kainic acid and pentylenetetrazole-induced seizures in rodents, an early short-term decrease in hippocampal VIP levels following the initial (precipitating) seizures was described (Marksteiner et al., 1989; Romualdi et al., 1992), suggesting that transient changes in VIP expression either contribute or counteract selective interneuron loss and plasticity changes during latent-period epileptogenesis. Preliminary studies *in vitro* show that changes in synaptic plasticity and synaptic plasticity markers following brief insults like hypoxia, bicuculine-induced seizures or inter-ictal like activity are either prevented or enhanced by a VPAC_1_ receptor antagonist, suggesting that different epileptogenic events are differentially regulated by VPAC_1_ receptor activity (Cunha-Reis, 2013; Carvalho-Rosa and Cunha-Reis, 2019).

In MTLE patients, the up-regulation of VIP receptors observed chronically is consistent with an increase in surviving neurons and levels of reactive glia (de Lanerolle et al., 1995; Clynen et al., 2014), suggesting that VPAC receptors (especially VPAC_1_) are promising targets for preventing epileptogenesis, a process that extends beyond the initial latent period (Devinsky et al., 2018). Given their role in the control of hippocampal synaptic plasticity they constitute also excellent candidates for prevention or attenuation of cognitive decline in MTLE. Furthermore, the dual role of VPAC_1_ and VPAC_2_ receptors in the control of hippocampal GABA release makes them the perfect targets for development of drugs aiming to control the imbalance in GABAergic and glutamatergic transmission associated with TLE (Schousboe et al., 2014; Cunha-Reis et al., 2017).

## Concluding Remarks

In conclusion, the importance of VIP, acting through VPAC_1_ or VPAC_2_ Rs, either to the control of hippocampal disinhibition leading to enhanced synaptic transmission or promoting a direct enhancement of pyramidal cell excitability suggests that VIP can have a differential impact in hippocampal-dependent cognition, and its possible therapeutic applications should be explored. The up-regulation of VIP receptors observed in MTLE patients and the finding obtained in animal models that the interneuron targets of VIP-containing interneurons are particularly susceptible to epileptic damage, suggest that VPAC receptors (especially VPAC_1_) are promising targets for epileptogenesis prevention and for prevention or attenuation of cognitive decline in MTLE.

## Author Contributions

AC-R: writing – review and editing. DC-R: resources, supervision, funding acquisition, project administration, and writing – original draft, review and editing.

## Conflict of Interest

The authors declare that the research was conducted in the absence of any commercial or financial relationships that could be construed as a potential conflict of interest.
