# Corrigendum: VIP Modulation of Hippocampal Synaptic Plasticity: A Role for VIP Receptors as Therapeutic Targets in Cognitive Decline and Mesial Temporal Lobe Epilepsy

**DOI:** 10.3389/fncel.2021.691978

**Published:** 2021-05-14

**Authors:** Diana Cunha-Reis, Ana Caulino-Rocha

**Affiliations:** ^1^BioISI - Biosystems and Integrative Sciences Institute, Faculdade de Ciências, Universidade de Lisboa, Lisbon, Portugal; ^2^Departamento de Química e Bioquímica, Faculdade de Ciências, Universidade de Lisboa, Lisbon, Portugal

**Keywords:** VIP, synaptic plasticity, interneurons, hippocampus, MTLE, cognition, VPAC1 receptors

In the original article, there was a mistake in Figure 1 as published. Circuits on the left side of the Figure moved down and stretched and do not represent what is described in the text for VIP O/A interneurons and their targets, OLM cells that should have their cell body in the stratum oriens. The corrected Figure 1 appears below.

**Figure 1:**
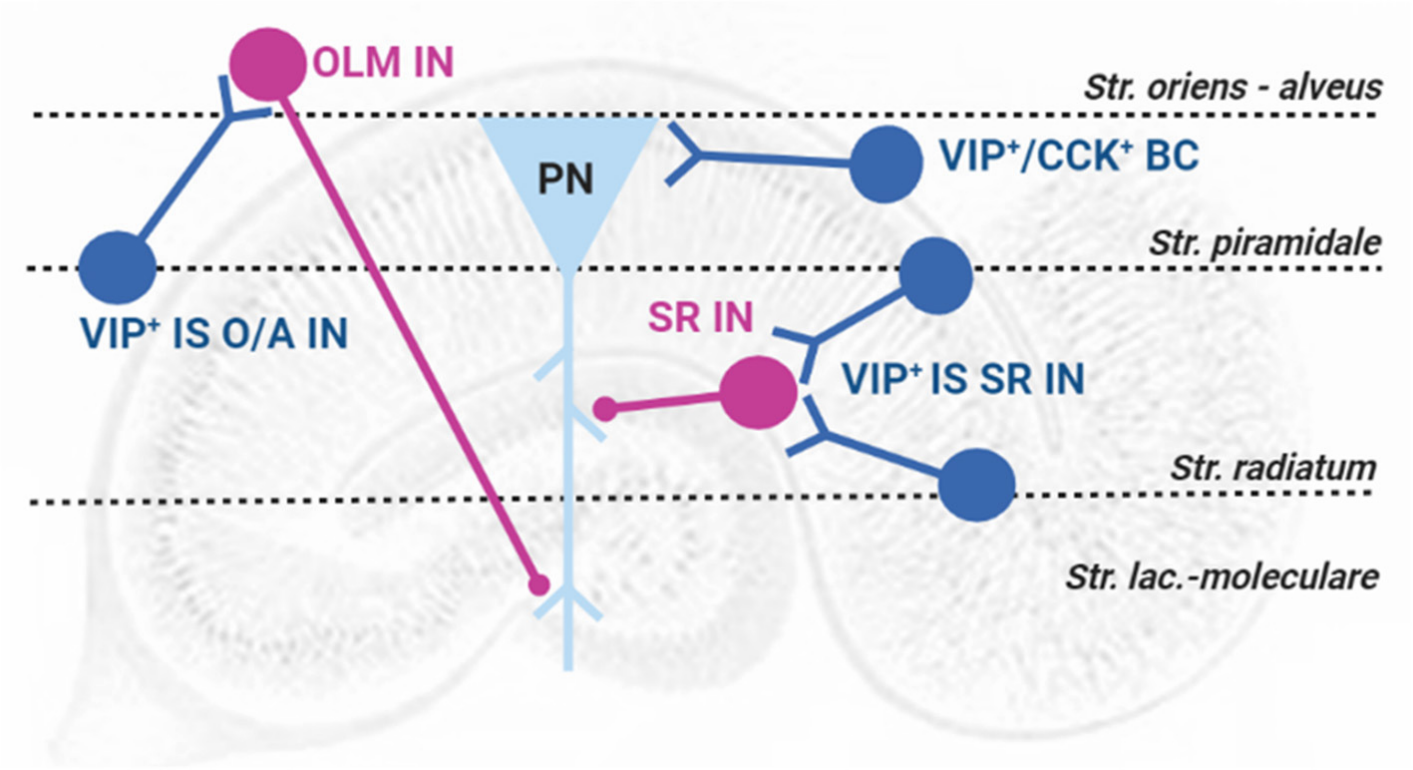
Representation of VIP-containing interneurons in the rat hippocampus: layer location and target selectivity. PN, pyramidal neuron (triangle, light blue); Interneurons (circles, pink); VIP-containing interneurons (circles, blue); *VIP*^+^*-CCK*^+^ *BCs*: VIP-containing *basket cells*; *VIP*^+^ *IS O/A IN*: VIP-containing interneuron-selective interneuron targeting the *stratum oriens*/*Alveus* and *VIP*^+^ *IS SR IN*: VIP-containing interneuron-selective interneuron targeting the *stratum radiatum*; OLM IN – *Stratum oriens* interneuron projecting to the *Stratum lacunosum-moleculare*; SR IN – *Stratum radiatum* local interneurons. Str.: *stratum*.

Additionally, in the original article, there was an error in the identification of type II IS cells (a different nomenclature for a subpopulation (and not all) of VIP+ interneurons projecting to the stratum radiatum).

A correction has been made to section *VIP in the Hippocampus*, Second *Paragraph*.

The corrected paragraph is shown below.

Detailed immunohistochemistry studies fully characterized hippocampal *VIP*^+^ *INs* dendritic trees and axon projections (Acsády et al., 1996a,b), allowing the classification of *VIP*^+^ *INs* into two fundamental groups according to their targets: *VIP*^+^ *basket cells* are responsible for somatic inhibition of pyramidal cells, are also immunoreactive for cholecystokinin (*VIP*^+^*-CCK*^+^ *BCs*, Figure 1) and do not express parvalbumin, as most *BCs* in the hippocampus. *VIP*^+^ *INs* that selectively innervate other interneurons (*VIP*^+^ *IS INs*) include two subtypes: (a) interneurons with cell bodies located at the *stratum pyramidale (SP)* or near and projecting to the *stratum Oriens/Alveus* border (*VIP*^+^ *IS O/A INs* or type III IS cells, Figure 1), that also express the interneuron marker calretinin and target mostly somatostatin-expressing (SOM^+^) *oriens lacunosum-moleculare* (*OLM*) interneurons innervating the distal dendrites of pyramidal cells at the *stratum lacunosum-moleculare (SLM)* and (b) *VIP*^+^ *INs* that project their axons to the *stratum radiatum* (*SR, VIP*^+^ *IS SR INs*, Figure 1), with cell bodies located either at the *SR/SLM* border (type II IS cells) or at *SR/SP* and targeting *interneurons* controlling synaptic transmission to proximal dendrites of pyramidal cells in the *SR* (Acsády et al., 1996a,b; Klausberger and Somogyi, 2008). In genetically modified VIP-eGFP mice, additional targets of *VIP*^+^ *IS O/A INs* in the *O/A*, including bistratified cells and oriens–oriens INs, have been described and recently a new VIP expressing interneuron population located at the O/A (*VIP*^+^ long-range projecting INs, *VIP*^+^ *LRP INs*) was described targeting *INs* within the O/A in CA1 but also both *INs* and pyramidal cells within the *subiculum* (Francavilla et al., 2018). It is not clear if it is also present in the rat hippocampus.

The authors apologize for these errors and state that they do not change the scientific conclusions of the article in any way. The original article has been updated.
